# Association between tobacco and alcohol use among young adult bar patrons: a cross-sectional study in three cities

**DOI:** 10.1186/1471-2458-14-500

**Published:** 2014-05-24

**Authors:** Nan Jiang, Youn Ok Lee, Pamela M Ling

**Affiliations:** 1 School of Public Health, The University of Hong Kong, 5/F William MW Mong Block, Room A5-08 21 Sassoon Road, Hong Kong, People’s Republic of China; 2 Public Health and Environment Division, RTI International, 3040 E. Cornwallis Road, P.O. Box 12194, Research Triangle Park, NC 27709, USA; 3 Center for Tobacco Control Research and Education and Division of General Internal Medicine, Department of Medicine, University of California, San Francisco, 530 Parnassus Avenue, Suite 366, San Francisco, CA 94143, USA

**Keywords:** Tobacco, Alcohol, Young adults, Bar patrons

## Abstract

**Background:**

Bars and nightclubs are key public venues where young adults congregate and use both tobacco and alcohol, and young adult bar patrons are at high risk for substance use. This study examined the association between cigarette smoking and alcohol use among a random sample of young adult bar patrons from three different cities in the USA.

**Methods:**

Cross-sectional data was collected from a random sample of young adult bar patrons aged 18–29 in San Diego, CA (N = 1,150), Portland, ME (N = 1,019), and Tulsa, OK (N = 1,106) from 2007–2010 (response rate 88%) using randomized time location sampling. Respondents reported the number of days they smoked cigarettes, drank alcohol, and binge drank in the past 30 days. Multinomial logistic regression was used to analyze the association between smoking (nonsmoker, occasional smoker, and regular smoker) and drinking and binge drinking for each city controlling for age, gender, race/ethnicity, and education. Predicted probabilities of each smoking category were calculated by drinking and binge drinking status. The association between smoking and drinking and binge drinking among combined samples was also analyzed, controlling for demographic variables and city.

**Results:**

Respondents reported high current smoking rates, ranging from 51% in Portland to 58% in Tulsa. Respondents in Tulsa were more likely to report regular smoking than those in San Diego and Portland, with demographic variables being controlled. Young adult bar patrons also exhibited a strong association between smoking and drinking. In general, as the frequency of drinking and binge drinking increased, the predicted probability of being a smoker, especially a regular smoker, increased in each city.

**Conclusions:**

Young adult bar patrons consistently reported a high smoking rate and a strong relationship between smoking and drinking, regardless of the different bar cultures and tobacco control contexts in each of the three cities. While smoke-free bar policies were negatively associated with regular smoking, these policies alone may not be enough to influence the association between smoking and drinking, particularly if tobacco marketing continues in these venues, or in the absence of programs specifically addressing the co-use of tobacco and alcohol.

## Background

Young adults smoke cigarettes at rates higher than any other age group [1]. According to the 2010 National Survey on Drug Use and Health survey, 34.2% of young adults aged 18 to 25 are current smokers, compared with 22.8% for adults aged 26 or older [1]. Cigarette smoking is strongly associated with alcohol consumption [2-8]. Studies of adolescents and young adults found that smokers (both daily and non-daily) were more likely than non-smokers to report alcohol consumption and binge drinking (five or more drinks per episode) [4,6]. Conversely, drinkers, especially binge drinkers, are more likely to smoke than non-drinkers [2,5]. The association between tobacco and alcohol use becomes stronger with the heavy use of either substance [3,9,10]. Smokers smoke more cigarettes while under the influence of alcohol [3,9,11,12], especially during binge drinking episodes [3,9].

Young adults may be particularly vulnerable to concurrent use of cigarettes and alcohol, and co-use of tobacco and alcohol may affect patterns of uptake of either or both substances. Young adults perceive an increased enjoyment of and desire for cigarettes while drinking alcohol [3,13]. Alcohol makes novice smokers feel it is physically easier to try smoking, and lowers inhibitions that limit cigarette consumption [14]. A study of young adult smokers found that 86% of experimenters’ and 63% of established smokers’ smoking episodes occurred with alcohol [3].

Bars and nightclubs are venues where young adults often congregate and use both tobacco and alcohol. Furthermore, bars and nightclubs are also the longstanding targets of aggressive tobacco marketing, much of which has focused on young adults [15,16] and featured alcohol by providing discounted or free alcohol and alcohol-related contests [17]. Therefore, young adults who frequent bars are at especially high risk for smoking. A previous study of young adult bar patrons in San Diego, California found that 47% of participants were current (past 30-day) smokers [18] while the state smoking prevalence was 12.5% among young adults aged 18 to 24 years [19]. A positive association between bar attendance and smoking has been found among young adults [13]. Thus, bar settings may encourage non-smokers to try cigarettes, smokers to smoke more cigarettes, and former smokers to relapse.

Although a positive association between tobacco and alcohol use has been documented in studies of young adults, these studies often focused on college students [3,5,20] who are less likely to smoke than those with lower education levels [6,18]. Few studies have examined the relationship between smoking and drinking among young adult bar patrons, a high risk population for substance use and are typically underrepresented in studies. The only study demonstrating the relationship between smoking and drinking in the young adult bar-going population used data collected in a single city, San Diego, CA, where a comprehensive tobacco control program has been established since 1989 [21-23] and a 100% smokefree law has been implemented for decades. Furthermore, adults in California report lower smoking rates than all other states except Utah [19]. Research is needed to better understand if the association between smoking and drinking that was observed is unique to a California population, or if it can be seen in other cities, particularly those with different smoke-free bar policies.

In the present study, we compared the association between smoking and drinking among young adult bar patrons in different cities. We obtained a random sample of young adult bar patrons using randomized time location sampling (TLS) strategies and collected data in three cities in three different regions of the US: San Diego, CA (West Coast), Portland, ME (Northeast), and Tulsa, OK (Midwest). By including both Portland and Tulsa in addition to San Diego, we were able to examine if the association between smoking and drinking was consistent across different cities with different cultural and tobacco control policy contexts.

## Methods

### Study design

Data collection procedures were reviewed and approved by the Committee on Human Research (the institutional review board) at the University of California San Francisco. Cross sectional randomly sampled data were collected in San Diego, CA, Portland, ME, and Tulsa, OK as part of efforts to evaluate young adult tobacco control programs in these cities. The cities were selected to leverage resources and investments made by the State health departments and other grant funding agencies to develop interventions for young adults in bars in these particular locations. Data for this study were collected prior to implementation of tobacco control programs to avoid contamination by intervention effects. The inclusion of cities in three different states included two sites in which bars had smokefree policies (San Diego and Portland) and one site with bars without smokefree policies (Tulsa).

Randomized time location sampling (TLS) strategies were used to access a random sample of the young adult bar-going population in each city. Randomized TLS has been widely used by public health professionals to collect data among hard-to-reach and “hidden” populations utilizing venues where the target populations tend to gather or congregate, such as men who have sex with men in certain bars and dance clubs, commercial sex workers in “red light” districts; or intravenous drug users at “shooting galleries” [24-27]. National surveillance surveys and censuses typically fail to obtain a large enough sample of “hidden” populations such as these. TLS addresses the challenges of accessing “hidden” populations, and approximates probability sampling methods and therefore, allows statistical inferences to a larger population [24-26]. In TLS, a census of all the specific venues, days, and time intervals the target population gathers is created. Venues, days, and times of data collection are randomly selected, and data are collected from either all or a sample of subgroup members present during these randomly selected time intervals. By randomly selecting venues, days, and times, members of the target population have approximately equal chances of being sampled, approximating probability sampling [24-26].

Informal qualitative interviews with 8–10 key informants, patrons in focus groups, and bar owners in each city were used to enumerate all bars and nightclubs popular among young adults (including the specific nights of the week and times of night), as well as local events, shows, and other venues frequented by young adults in each geographic location. Survey data collection venues and times were randomly selected from this list. Permission to collect data was obtained from bar managers at every venue, and bar entry fees were paid, when applicable.

Trained study personnel visited the selected locations at the designated randomly selected times, counted the total number of people present in the sampling area during the data collection period, and approached all individuals present at the time of sampling who appeared to be in the 18-29-year-old age range. Participants whose self-reported age was between 18–29 were invited to complete paper-and-pencil surveys. Trained personnel explained the study, and all participants verbalized that they understood they were participating in a voluntary research study. Verbal informed consent was utilized to maximize convenience for the participants. Patrons who agreed to participate in the study filled the questionnaire in the main bar, and, if needed, participants would temporarily step away from friends while completing the questionnaire to avoid being disturbed. On average, it took about 10–15 minutes for the participants to complete the survey. As a fidelity check, trained “secret shoppers” visited the selected venues at times unknown to the survey teams to observe and interact with them to ensure the teams were following protocols for randomization, verbal informed consent, and approaching all eligible patrons. Reports from secret shoppers were also used to provide feedback to survey teams. Patrons who appeared to be intoxicated or who were unable or unwilling to complete the verbal informed consent procedure for any reason were not included. Detailed study information was offered to all participants in an information sheet, and all participants were given a study business card containing contact information for the study, and a link to the study website, which also contained a copy of the informed consent form. After surveys were collected, age was cross-checked using date of birth. Only respondents aged 18–29 by date of birth were included in data analysis.

Bars open and close frequently, and a venue’s popularity also changes. Therefore, the list of target bars and times was updated every two weeks so that newly opened bars were included and bars that closed were eliminated from the list. In addition, bars that were found not to match the desired target when visited for data collection (e.g., the patrons present were all too old) were eliminated from the list during the following data collection period, in accordance with the TLS protocol [24]. The specific times for data collection at each particular location were enumerated, based on the site’s popularity among young adults at that time of night. Most data collection took place in a two-hour time slot (e.g., 9–11 PM). Because of young adult venue patronage patterns, for all sites, all of the data collection in this study was completed between 8 PM and 12 AM. Utilizing this method, 1,150 surveys were collected in San Diego between December 2007 and February 2008, 1,019 in Portland between October and November 2008, and 1,106 in Tulsa between January and March 2010. Across all sites, an average of 88% of those within the age range invited to participate agreed to complete surveys, ranging from 86% in Portland and Tulsa to 92% in San Diego.

### Main measures

Participants reported the number of days in the past 30 days they smoked at least one cigarette. Responses were coded as: 0 = non-smokers (0 days), 1 = occasional smokers (< 20 days), and 2 = regular smokers (≥ 20 days). Because there are no universal standard definitions of frequency of smoking and frequency of drinking or binge drinking in the literature, respondents were categorized into groups based on the frequency distribution of their responses. Specifically, 45% of samples were non-smokers. Half of smokers reported smoking less than 20 days in past month, and another half smoked ≥ 20 days. This definition is consistent with that used in our prior studies of young adult bar patrons, where smoking 20 or more days per month is considered to be regular smoking [18]. Participants also reported the number of days they drank any alcohol in the past 30 days, and were categorized into non-drinkers (0 days), occasional drinkers (< 10 days), and frequent drinkers (≥ 10 days) based on the frequency distribution (33% of respondents reported alcohol consumption on < 10 days and 63% reported drinking on ≥ 10 days in past month). The same cutoff of ≥ 10 days for frequent drinking has been used in a previous study of young adults [5]. Similarly, binge drinking (at least five shots/drinks in the same night) was categorized into no binge drinking (0 days), occasional binge drinking (< 10 days), and frequent binge drinking (≥ 10 days) (47% of respondents reported binge drinking on < 10 days and 31% reported binge drinking on ≥ 10 days in past month). Demographics included gender, age, race/ethnicity, and education.

### Statistical analysis

Multinomial logistic regression models were used to analyze the association of smoking (0 = non-smoker, 1 = occasional smoker, 2 = regular smoker) with drinking and binge drinking for each city, controlling for age, gender, race/ethnicity, and education, using STATA version 11.0. We conducted multinomial regression instead of ordinal logistic regression, because ordered logistic regression models assume that the distance between each category of the outcome is proportional. Although smokers were categorized by the number of smoking days, we cannot assume that the distance between nonsmoker, occasional smoker, and regular smoker categories are the same. Predicted probabilities of each smoking category were calculated by drinking and binge drinking status, using the prtab command [28]. Then the samples from the three cities were combined, and a multinomial logistic regression model was used to examine the association between smoking and drinking and binge drinking, controlling for age, gender, race/ethnicity, education, and city. To assess if the association between smoking and drinking differs by city, the Bayesian information criterion (BIC) test was conducted for two models: a full model included drinking, city, and the interaction effect of the drinking and city as independent variables, controlling for age, gender, race/ethnicity, education; and a restricted model which dropped the interaction variable. The restricted model produced a smaller BIC, indicating the restricted model was a better-fitting model. The same test was also conducted to examine the interaction effect of binge drinking and city. The result showed that the restricted model produced a smaller BIC than the full model. Therefore, we dropped the interaction factors from the multinomial logistic regression model and report findings from the restricted model only.

## Results

**Figure 1:**
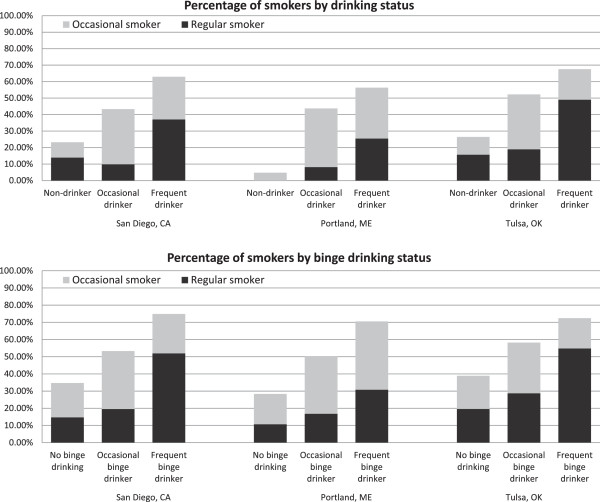
**Percentage of samples in each smoking category by drinking and binge drinking status.***Note.* Occasional smokers smoked on 1–19 of the past 30 days; regular smokers smoked on ≥ 20 days in the past 30 days. Occasional drinkers drank alcohol on 1–9 of the past 30 days; frequent drinkers drank on ≥ 10 days in the past 30 days. Occasional binge drinkers reported binge drinking on 1–9 of the past 30 days; frequent binge drinkers reported binge drinking on ≥ 10 days in the past 30 days.

In general, the samples were evenly split between males and females, and the mean age was approximately 23 years old (Table 1). Race/ethnicity reflected the population in the different cities, with more Hispanics and Asians in San Diego, CA than the other sites. Approximately 30-40% of respondents were college graduates, 40% were currently in college, with 20-30% reporting high school education only or dropping out of college. Young adult bar patrons displayed high rates of current smoking, ranging from 51% in Portland to 58% in Tulsa. Among smokers, 38% were regular smokers in Portland, 51% in San Diego, and 59% in Tulsa.Figure 1 shows the percentage of young adult bar patrons in each smoking category by drinking and binge drinking status. About 43-52% of occasional drinkers and 56-68% of frequent drinkers smoked, and 50-58% of occasional binge drinkers and 71-75% of frequent binge drinkers smoked. The smoking and regular smoking rate generally increased as the frequency of drinking and binge drinking increased. Among each drinking and binge drinking group, young adults in Tulsa generally exhibited the highest smoking and regular smoking rates, and Portland showed the lowest rates.

**Table 1:** Sample characteristics and prevalence of tobacco and alcohol use

	**San Diego, CA (n = 1150) %**	**Portland, ME (n = 1019) %**	**Tulsa, OK (n = 1106) %**	**Total (N = 3275) %**
Mean age (SD)	23.7 (1.96)	23.0 (1.83)	23.1 (2.43)	23.3 (2.11)
Gender				
Male	50.6	56.1	54.9	53.3
Female	49.4	43.9	45.1	46.7
Race/Ethnicity				
White	61.1	69.9	70.7	67.1
Black	4.2	7.6	4.2	5.2
Asian/Pacific Islander	9.0	7.4	2.0	6.1
Hispanic	14.2	8.2	7.4	10.1
Others	11.5	7.0	15.7	11.5
Education				
High school	6.5	11.6	15.1	11.0
Dropped out college	12.2	10.4	13.5	12.1
In college	42.9	39.4	40.0	40.8
Graduated from college	38.4	38.5	31.4	36.1
Smoking status^a^				
Non-smoker	43.9	48.6	42.0	44.7
Occasional smoker	27.5	32.0	23.7	27.6
Regular smoker	28.6	19.4	34.3	27.7
Drinking status^b^				
Non-drinker	3.8	2.1	7.7	4.6
Occasional drinker	27.5	31.5	38.7	32.5
Frequent drinker	68.7	66.4	53.7	63.0
Binge drinking^c^				
No binge drinking	21.6	23.1	21.9	22.2
Occasional binge drinking	46.8	47.1	47.9	47.3
Frequent binge drinking	31.6	29.9	30.2	30.6

Note.

^a^Occasional smokers smoked on 1–19 of the past 30 days; regular smokers smoked on ≥ 20 days in the past 30 days.

^b^Occasional drinkers drank alcohol on 1–9 of the past 30 days; frequent drinkers drank on ≥ 10 days in the past 30 days.

^c^Occasional binge drinkers reported binge drinking on 1–9 of the past 30 days; frequent binge drinkers reported binge drinking on ≥ 10 days in the past 30 days.

The predicted probabilities of young adults’ being a smoker, particularly a regular smoker, increased as the frequency of drinking and binge drinking increased for each city, holding age, gender, race/ethnicity, and education at their mean values (Table 2). For each drinking and binge drinking group, young adult bar patrons in Tulsa showed the highest predicted probabilities of regular smoking, holding covariates at their mean values, except for frequent binge drinkers who showed a higher predicted probability of being a smoker in San Diego (0.74) than in Tulsa (0.72).

**Table 2:** Predicted probability of cigarette smoking by drinking and binge drinking status

	**San Diego, CA**				**Portland, ME**				**Tulsa, OK**			
	**Non smoker**	**Occasional smoker**^**a**^	**Regular smoker**^**b**^	**Total**	**Non smoker**	**Occasional smoker**	**Regular smoker**	**Total**	**Non smoker**	**Occasional smoker**	**Regular smoker**	**Total**
Drinking status^c^												
Non-drinker	.81	.07	.12	**1.00**	.96	.04	.00	**1.00**	.77	.11	.13	**1.00**
Occasional drinker	.56	.34	.10	**1.00**	.57	.35	.08	**1.00**	.48	.34	.18	**1.00**
Frequent drinker	.38	.26	.36	**1.00**	.44	.30	.25	**1.00**	.32	.19	.49	**1.00**
Binge drinking^d^												
No binge drinking	.66	.19	.15	**1.00**	.74	.17	.10	**1.00**	.62	.20	.18	**1.00**
Occasional binge drinking	.46	.34	.20	**1.00**	.50	.33	.17	**1.00**	.41	.30	.28	**1.00**
Frequent binge drinking	.26	.23	.51	**1.00**	.30	.39	.31	**1.00**	.27	.18	.54	**1.00**

Note.

^a^Respondents who smoked cigarettes on 1–19 days in the past 30 days.

^b^Respondents who smoked cigarettes on ≥ 20 days in the past 30 days.

^c^Occasional drinkers drank alcohol on 1–9 of the past 30 days; frequent drinkers drank alcohol on ≥ 10 days in the past 30 days.

^d^Occasional binge drinkers reported binge drinking on 1–9 of the past 30 days; frequent binge drinkers reported binge drinking on ≥ 10 days in the past 30 days.

Predicted probabilities calculated based on results from multinomial logistic regression. Predicted probabilities are shown for each drinking and binge drinking category, with all demographic variables held constant at their mean values, including age, gender, race/ethnicity, and education.

Occasional drinkers were more likely to report occasional smoking than non-drinkers, and frequent drinkers were more likely to be occasional and regular smokers, controlling for age, gender, education, race/ethnicity, city and binge drinking status (Table 3). Also both occasional and frequent binge drinkers reported greater likelihood of being smokers with demographics, city and drinking status being controlled. College students, those who dropped out of college and high school graduates were more likely to report regular smoking than college graduates, controlling for demographics, city, drinking and binge drinking status. Young adults in San Diego and Portland were less likely to be regular smokers than those in Tulsa.

**Table 3:** Predictors of cigarette smoking among young adult bar patrons

	**Occasional smoker**^**a**^	**Regular smoker**^**b**^
	**AOR [95% CI]**	**AOR [95% CI]**
Age	0.94 [0.90, 0.99]^*^	0.94 [0.90, 0.99]^*^
Gender		
Male	1.00	1.00
Female	0.93 [0.78, 1.11]	0.98 [0.81, 1.19]
Education		
College graduates	1.00	1.00
College students	1.16 [0.94, 1.43]	1.28 [1.02, 1.61]^*^
Dropped out college	1.51 [1.11, 2.06]^*^	2.86 [2.11, 3.88]^***^
High School	1.29 [0.92, 1.80]	2.80 [2.04, 3.86]^***^
Race/ethnicity		
White	1.00	1.00
Black	1.00 [0.67, 1.50]	0.93 [0.61, 1.42]
Asian/Pacific Islander	1.31 [0.92, 1.88]	1.15 [0.77, 1.72]
Hispanic	1.58 [1.19, 2.09]^**^	0.84 [0.60, 1.17]
Others	1.46 [1.09, 1.94]^*^	1.40 [1.05, 1.89]^*^
Location		
Tulsa, OK	1.00	1.00
San Diego, CA	1.07 [0.85, 1.35]	0.72 [0.58, 0.91]^**^
Portland, ME	1.07 [0.86, 1.34]	0.41 [0.32, 0.53]^***^
Drinking status^c^		
Non-drinker	1.00	1.00
Occasional drinker	3.73 [1.97, 7.10]^***^	1.36 [0.76, 2.41]
Frequent drinker	2.99 [1.55, 5.74]^**^	3.64 [2.05, 6.47]^***^
Binge drinking^d^		
No binge drinking	1.00	1.00
Occasional binge drinking	2.15 [1.68, 2.75]^***^	1.66 [1.25, 2.21]^***^
Frequent binge drinking	3.15 [2.33, 4.26]^***^	4.08 [2.99, 5.56]^***^

Note.

AOR = adjusted odds ratio; CI = confidence interval.

^a^Respondents who smoked cigarettes on 1–19 days in the past 30 days.

^b^Respondents who smoked cigarettes on ≥ 20 days in the past 30 days.

^c^Occasional drinkers drank alcohol on 1–9 of the past 30 days; frequent drinkers drank alcohol on ≥ 10 days in the past 30 days.

^d^Occasional binge drinkers reported binge drinking on 1–9 of the past 30 days; frequent binge drinkers reported binge drinking on ≥ 10 days in the past 30 days.

^*^P < .05; ^**^P < .01; ^***^P < .001.

## Discussion

Young adult bar patrons reported a high current smoking rate. More than half of the young adults surveyed had smoked in past month. The smoking rate among young adult bar patrons was higher than the state young adult smoking prevalence in the three sites studied. In San Diego in 2007–2008, bar patrons aged 18–29 and 18–24 (data not shown in tables) reported smoking rates of 56% and 59% respectively, which was considerably higher than the young adult (aged 18–24) smoking rate of 15% in 2007 and 17% in 2008 reported by the Behavioral Risk Factor Surveillance System [19]. Young adult bar patrons aged 18–29 and 18–24 in Portland respectively reported smoking rates of 51% and 52% in 2008, compared to the state young adult aged 18–24 smoking prevalence of 28% in 2008 [19]. In Tulsa, 58% of young adult bar patrons aged 18–29 and 59% of bar patrons aged 18–24 reported smoking in 2010; the state young adult (aged 18–24) smoking prevalence was 28% in 2010 [19]. Prior studies have noted disproportionately high smoking rates among bar patrons, and that smoking status was strongly associated with bar attendance [13]. The findings suggest that young adults attending bars and nightclubs are at high risk for smoking, and that tobacco control programs for young adults should prioritize interventions in social entertainment venues frequented by high risk young adults.

Young adult bar patrons exhibited a strong association between cigarette smoking and alcohol use in each of the three cities. As the frequency of drinking and binge drinking increased, the predicted probability of being a smoker increased. This association was even stronger than observed in prior studies. Among young adult bar patrons, 50-58% of occasional binge drinkers and 71-75% of frequent binge drinkers from the three cities smoked; whereas in a 2001 study of a national representative sample of American college students, Weitzman and Chen [5] found that 44% of college students who reported binge drinking on at least one day in the past month smoked. In addition, among young adult bar patrons, 68% of frequent drinkers in Tulsa smoked, whereas among college students, 57% of frequent drinkers smoked [5]. These differences between young adult bar patrons and college student samples indicate that young adult bar patrons might more strongly associate cigarette smoking and alcohol use, than college students in general, although the data are not directly comparable since our bar patron data were collected several years later than Weitzman and Chen’s study.

One potential reason for the strong association between smoking and drinking among young adult bar patrons is that, in addition to the rewarding effects of nicotine and alcohol [10,11], tobacco marketing efforts in bars frequently linked tobacco and alcohol use and targeted young adults [15-17]. Although smokefree bar policies have been implemented in some places (e.g., California implemented smokefree bar laws in 1998), tobacco industry bar promotions continue. For years, the California Department of Health has received reports from tobacco companies like Philip Morris detailing where events were planned to take place in California (including specifically in San Diego) in smokefree bars, continuing through 2014. These tobacco marketing activities at bars and nightclubs may reinforce the strong link between tobacco and alcohol use among bar patrons. Furthermore, loopholes exist in the current smokefree bar laws. For example, smoking is allowed in outdoor areas at the bars and nightclubs, and California’s smokefree laws exempt sole proprietor businesses, and therefore, smoking is still allowed in a number of bars. Novel tobacco control measures such as policies prohibiting tobacco promotions in venues where alcohol is served or sold, including adult-only venues like bars and nightclubs, and smokefree outdoor policies may help reduce smoking among bar patrons. This is a cross sectional study and so cannot address causality in the observed associations. In fact, it is plausible that a bidirectional relationship between drinking and smoking episodes exists, where increased smoking may facilitate increased alcohol consumption, and increased alcohol consumption may also lead to increased smoking. Tobacco cessation programs for young adult smokers should address the pairing of tobacco and alcohol use, and educate young adults about the risks of paired use.

College students were more likely to be regular smokers than those who had graduated from college. One potential explanation for this pattern might be that, for many college students, the transition from high school to college may include establishing a new social network which often involves parties on campus and in bars and nightclubs. At these parties, college students may smoke to reinforce an identity of being cool and in control for males or being fun and outgoing for females. Therefore, for college students, smoking patterns might be linked strongly to social behavior at parties coupled with the desire to project a specific identity. These motivations or social contexts may be less important after graduation, so that the college graduates are less likely to report regular smoking than college students.

Young adult bar patrons in Tulsa were more likely to report smoking, especially regular smoking than their counterparts in San Diego and Portland. One potential explanation might be that San Diego and Portland have implemented smokefree bar policies, while Tulsa has not. Prior studies have demonstrated that smokefree bar policies are an effective means of protecting non-smokers (both bar staff and patrons) from secondhand smoke, reducing smoking rates, increasing smokers’ desire to quit, and increasing their likelihood of cessation [29-33]. As smokefree bar policies send out the message that smoking is not socially normative and acceptable in bars and make it inconvenient to smoke, smokers may reduce smoking when out drinking in smokefree bars. The strength of the association between smoking and drinking or binge drinking was not affected by location. These results suggest that while smokefree bar policies may reduce regular smoking prevalence, the association between smoking and drinking may persist amongst bar patrons even after smoke-free policy implementation. A larger sample of localities with different smokefree bar policies would be a logical next step to examine the impact of bar policies on the association between tobacco and alcohol use.

This study should be interpreted in light of the following limitations. First, data were collected in three cities. About 41% of our sample were current college students and 36% were college graduates. The proportion of young adults with higher education is greater than the general young adult population, and probably reflects the educational status of young adults attending college bars, which were included in the sample. Thus, findings may not be generalized to the entire young adult bar-going population across the nation. However, the utilization of randomized time location sampling strategies allowed a random sample of young adult bar patron population. Second, as cities were selected to leverage resources and investments made by the state health departments and grant funding agencies for a separate intervention project, data were collected (prior to intervention) from locales which might be unparallel in terms of smoking prevalence and other conditions, and differences in funding streams caused data to be collected at slightly different times. Thus, the observed differences in the association between tobacco and alcohol use among the three cities might be associated with some uncontrolled conditions. Third, the self-reported past-month smoking and drinking behaviors included the simultaneous use of cigarettes and alcohol at the same episode, as well as the use of either substance at separate occasions. We did not attempt to capture the temporal association between smoking and drinking in this study. Despite this limitation, there was a consistency in the strong association between smoking and drinking across sites and at different times. The present study also used sample-customized smoking classifications as dependent variable categories, and used multinomial logistic regression models to examine the association between smoking and alcohol use, thus allowing a more appropriate categorization of cigarette smoking behavior for this population. Fourth, like all studies using questionnaires, respondents might misreport their behaviors. Therefore, results were subject to measurement errors.

## Conclusions

This study contributes to an emerging body of literature focused on the high smoking rates among young adults. By using randomized time location sampling this study used an innovative method to access the hard-to-reach and under-studied population of young adult bar patrons. This population is at heightened risk for smoking and drinking and improves upon prior studies limited to the college student population [3,5,20]. Furthermore, these results go beyond those of prior studies of bar attendance to compare data on tobacco and alcohol use from multiple cities with different smokefree bar policies. We found a consistent high smoking rate and strong association between smoking and drinking in all cities, regardless of their bar cultures and tobacco control contexts. Smoke-free bar policies alone may not be enough to influence the association between smoking and drinking, particularly if tobacco marketing continues in these venues, or in the absence of programs specifically addressing co-use of tobacco and alcohol. Tobacco interventions should prioritize bars and other social venues popular among young adults to reach those at greatest risk. The strong and consistent association between smoking and drinking indicates that public health efforts and clinical cessation programs need to address the paired use of tobacco and alcohol among the young adult bar-going population.

## Competing interests

The authors declare that they have no competing interests.

## Authors’ contributions

NJ designed the study, analyzed the data, and led the writing of the manuscript. YOL assisted in data analysis and interpretation of findings. PML conceptualized and supervised the study. All authors revised the manuscript critically, and approved the final manuscript.

## Pre-publication history

The pre-publication history for this paper can be accessed here:

http://www.biomedcentral.com/1471-2458/14/500/prepub
